# Cystoid Macular Edema in a 10-Year-Old Boy With Cohen Syndrome

**DOI:** 10.7759/cureus.8443

**Published:** 2020-06-04

**Authors:** Caleb A Liles, Michael S Tensmeyer, Justin M York, Lakmal S Ekanayake, Julie Lew

**Affiliations:** 1 Ophthalmology, Ohio University Heritage College of Osteopathic Medicine, Athens, USA; 2 Neuroscience, Ohio University Heritage College of Osteopathic Medicine, Athens, USA; 3 General Surgery, Ohio University Heritage College of Osteopathic Medicine, Athens, USA; 4 Medicine, Ohio University Heritage College of Osteopathic Medicine, Athens, USA; 5 Ophthalmology, Holzer Health System, Athens, USA

**Keywords:** cohen syndrome, chorioretinal dystrophy, retina, retinitis pigmentosa

## Abstract

Cohen syndrome is an extremely rare disease with characteristic somatic and multi-system features that severely affect vision. Ophthalmologists must consider Cohen syndrome when developmental delay, high-grade myopia, and retinal dystrophy are present in a child. Here we report a case of Cohen syndrome in a 10-year-old boy presenting with cystoid macular edema (CME), only the second reported case of its kind. This case illustrates the phenotypic variability that can occur in Cohen syndrome, with rare features in addition to CME including trace posterior subcapsular cataracts, growth hormone deficiency, mild vermian hypoplasia, a nasolacrimal cyst, hearing loss, and high-functioning intelligence quotient (IQ). Our patient did not have an identifiable second mutation even after extensive genetic testing, which raises questions about whether the patient has a novel gene variant for the disease or an autosomal dominant mode of inheritance exists for Cohen syndrome. In addition to peripheral vision loss, the rare appearance of macular edema can threaten the remaining vision and requires intervention. This case also demonstrates that, without a high index of suspicion, there can be considerable delay in diagnosing Cohen syndrome. Though little is known about the prevalence of many of the clinical features seen in our case in the Cohen syndrome population, this case raises awareness of the syndrome and the need to recognize various clinical features, perform genetic testing, and direct appropriate treatment to prevent complications and help improve quality of life.

## Introduction

Cohen syndrome is an extremely rare autosomal recessive disorder described in less than 1,000 individuals. This syndrome is characterized by somatic features, intellectual disability, neutropenia, and ophthalmic abnormalities. Facial features include microcephaly, long eyelashes, downslanting or wave-shaped palpebral fissures, a short, upturned philtrum, and a grimacing smile. Other somatic features include truncal obesity, hypotonia, and joint hyperextensibility [1,2]. The vast majority of individuals with Cohen syndrome are noted to have a cheerful disposition, feeding difficulties in the neonatal period, and delayed onset of puberty. Many ophthalmic findings have been reported in Cohen syndrome, with high-grade myopia and chorioretinal dystrophy most common [3,4]. Cohen syndrome has an increased incidence in Ohio Amish and Finnish populations [2].

The pathogenesis of Cohen syndrome has its origins in the Golgi complex. An abnormal vacuolar protein sorting 13 homolog B (VPS13B) gene leads to glycosylation defects in the Golgi, resulting in the accumulation of abnormal protein products. VPS13B, a transmembrane protein involved in protein sorting, has critical functions in the eye, blood, central nervous system, and adipocyte development [5-7].

The diagnosis of Cohen syndrome is mainly clinical, although confirmation by genetic testing may be necessary if the clinical features are unclear. Highly sensitive and specific clinical criteria, such as those proposed by Chandler et al. have been developed to aid in diagnosis [8]. These criteria are described in the Discussion section.

This case report mainly focuses on the ophthalmologic manifestations of Cohen syndrome. High-grade myopia and progressive retinal dystrophy leading to constricted visual fields usually occur by the second decade of life [3]. The retinal dystrophy in Cohen syndrome resembles retinitis pigmentosa and, as seen in this case, this crossover can delay the diagnosis. Similar to retinitis pigmentosa, Cohen syndrome displays nyctalopia and visual field constriction that can be the initial complaints that lead concerned parents to have their child evaluated. 

Examples of ophthalmologic findings that have been reported in Cohen syndrome include microphthalmia, microcornea, bull’s eye maculopathy, optic atrophy, cortical lens opacities, lens subluxation, wave-shaped palpebral fissures, coloboma and acute angle-closure glaucoma. Most commonly, vision becomes limited to counting fingers and light perception [9]. Management of the ophthalmologic manifestations generally relies on refractive correction with glasses, low vision training and annual comprehensive evaluation to monitor for progression of retinal dystrophy and appearance of other ocular complications that may arise.

For the primary care physician, the management of these patients is mainly supportive with psychosocial counseling, genetic counseling and ophthalmology referrals. Physical, occupational and speech counseling can help address issues related to developmental delays and motor functioning. As these patients tend to have recurrent infections from neutropenia, complete blood counts should be done annually and granulocyte-colony stimulating factor (G-CSF) therapy can be used to restore neutrophil counts [2]. 

Here we report a case of Cohen syndrome presenting with CME. To our knowledge, only one other case of CME has been reported in this syndrome [10].

## Case presentation

A 10-year-old boy presented to a retina specialist with symptoms of nyctalopia and decreased vision. He was referred by an optometrist after an evaluation that included an abnormal optical coherence tomography (OCT) scan. He was followed since the age of two by the optometrist and his examination was limited to refractive evaluation prior to this scan. His parents became concerned when he could not see objects directly in front of him and he frequently ran into household objects. They noted that he even ran into a tree while at a birthday party at the age of six, and has been followed by his primary care physician for multiple complaints. This history prompted further workup by the optometrist.

The patient had a relatively complex medical history, starting during gestation. During pregnancy, his mother had oligohydramnios and decreased fetal movements. At birth, the patient had joint laxity with associated hypotonicity. Shortly after an in-hospital birth, he required resuscitation due to a nasolacrimal cyst obstructing his airway, which was subsequently removed. Both physical and mental development were delayed, as he did not sit until eight months of age, the cruise until 14 months, and walk until 17 months. He had a history of growth hormone (GH) deficiency and delayed bone age, which, after discussion of the risks and benefits with the parents, were not treated with GH replacement. MRI of the brain at age two for GH deficiency revealed mild vermian hypoplasia among other non-specific findings. He had also had frequent illnesses, particularly ear infections with high fevers. He recently failed his hearing test at school but had not yet had a formal hearing evaluation. 

**Figure 1:**
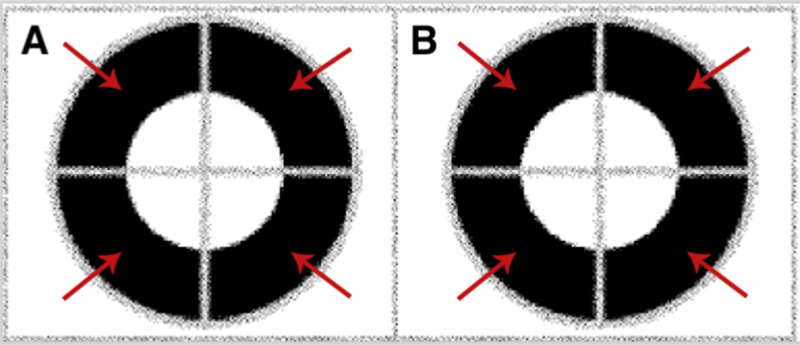
Informal illustration of visual field testing Visual fields demonstrate peripheral constriction bilaterally, as indicated by the red arrows. A: right eye (OD), B: left eye (OS)

**Figure 2:**
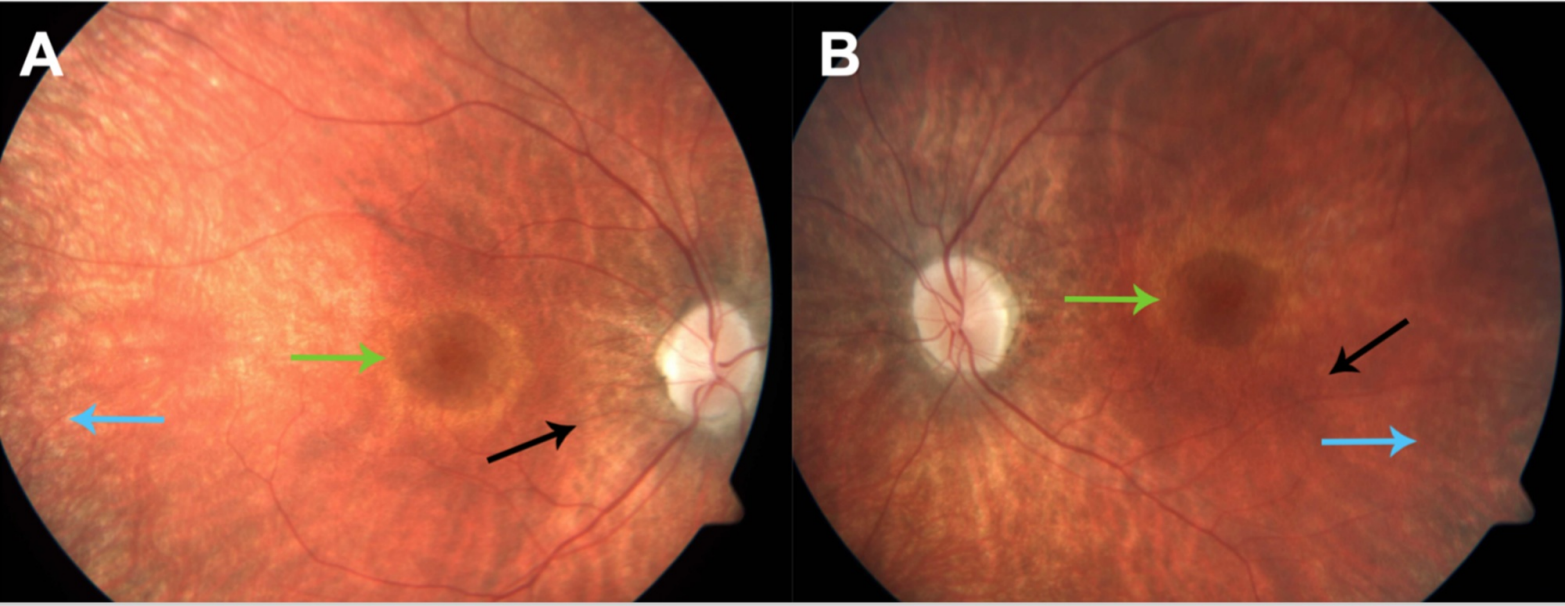
Fundus photos Fundus photos demonstrate retinal vessels with obvious (2+) arteriolar attenuation (black arrows), parafoveal pigmentation with surrounding pale regions (“bulls-eye” maculopathy - green arrows), and diffuse retinal pigment epithelial mottling peripherally (blue arrows). A: right eye (OD), B: left eye (OS)

**Figure 3:**
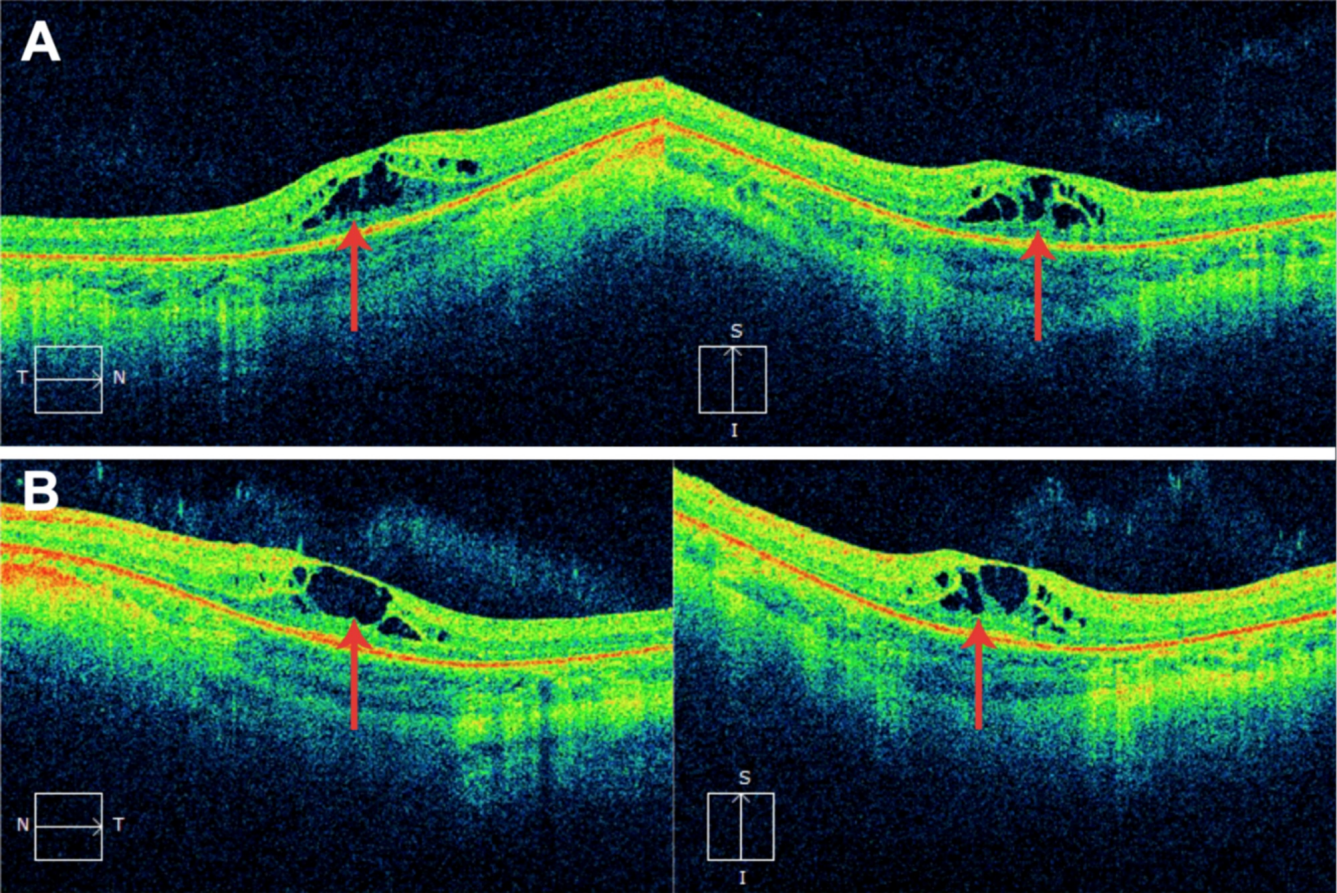
Optical coherence tomography (OCT) OCT demonstrates cystoid intraretinal fluid pockets primarily in the outer plexiform layer of the macula bilaterally, as indicated by the red arrows. Images on the left depict axial cross-sections, while images on the right depict longitudinal cross-sections of the respective eye. A: right eye (OD), B: left eye (OS)

On physical examination, visual acuity was 20/50 right eye (OD) and 20/40 left eye (OS) with an intraocular pressure (IOP) of 18 and 19, respectively. His refractive error was -2.25 OD and -2.50 OS. An informal illustration of visual field testing is shown in Figure 1. Blunted focal light reflexes were noted bilaterally. Trace posterior subcapsular cataracts were also noted, along with 1+ cells in the vitreous and optic nerve pallor. The cup-to-disc ratio was 0.2. The retinal vessels had 2+ arterial attenuation, parafoveal pigmentation with “bulls-eye” maculopathy, and diffuse retinal pigment epithelial mottling peripherally, as seen in Figure 2. OCT showed CME bilaterally, for which dorzolamide 2% gtt t.i.d. was prescribed (Figure 3). 

Approximately six months after evaluation by a retina specialist and completion of a retinal dystrophy panel, the patient was referred to a pediatric genetic ophthalmologist. During the evaluation, the genetic pediatric ophthalmologist noted the patient's somatic features. The patient had downslanting palpebral fissures, a short upturned philtrum with a grimacing smile, and long eyelashes. Other somatic features included truncal obesity, hypotonia, and joint hyperextensibility. Despite previous delays, the patient had a period of catch-up growth and was intellectually high-functioning. Intelligence quotient (IQ) and other cognitive functions were intact and normal.

Initial genetic studies showed a single gene variant of undetermined significance associated with Cohen syndrome. A more thorough evaluation to detect a second mutation including deletion/duplication variants was ordered, but was negative.

## Discussion

Several features of this case are shared with the limited available case reports of patients with Cohen syndrome, including cystoid macular edema (CME), trace posterior subcapsular cataracts, GH deficiency, mild vermian hypoplasia, a nasolacrimal cyst, hearing loss, and high-functioning IQ. Our patient also had 1+ vitreous cells and optic disc pallor on examination, both of which are associated with CME. One other case of CME in Cohen syndrome has been reported, which was a genetically confirmed VPS13B homozygous mutation case with bilateral CME [10]. 

The fact that our patient does not have a known second mutation for Cohen syndrome certainly raises questions regarding the known mechanisms and gene variants of the disease. Either there is an autosomal dominant mechanism in addition to the already known autosomal recessive inheritance, or more likely the patient has a novel gene variant for the second mutation. In either case, this represents a significant finding for our knowledge of the disease. Current research is being performed to identify gene variants that exist in the parents as well as the siblings, if present. 

**Figure 4:**
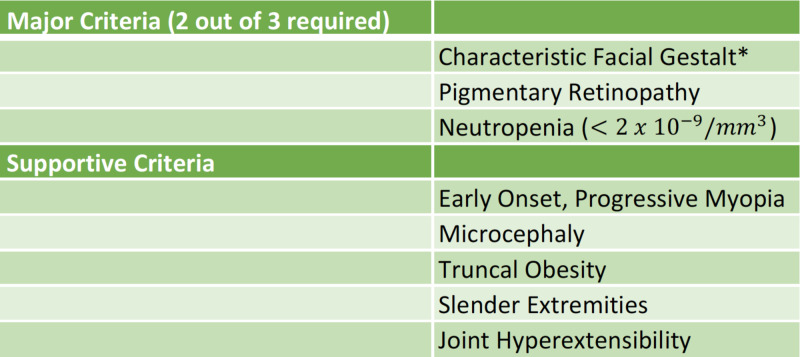
Major and minor criteria for diagnosis of Cohen syndrome The figure lists the diagnostic criteria for Cohen syndrome, in the context of a child with significant learning disabilities. *Includes thick hair, eyebrows and eyelashes, wave-shaped downward slanting palpebral fissures, prominent beak-shaped nose, short upturned philtrum with the grimacing expression on smiling

**Figure 5:**
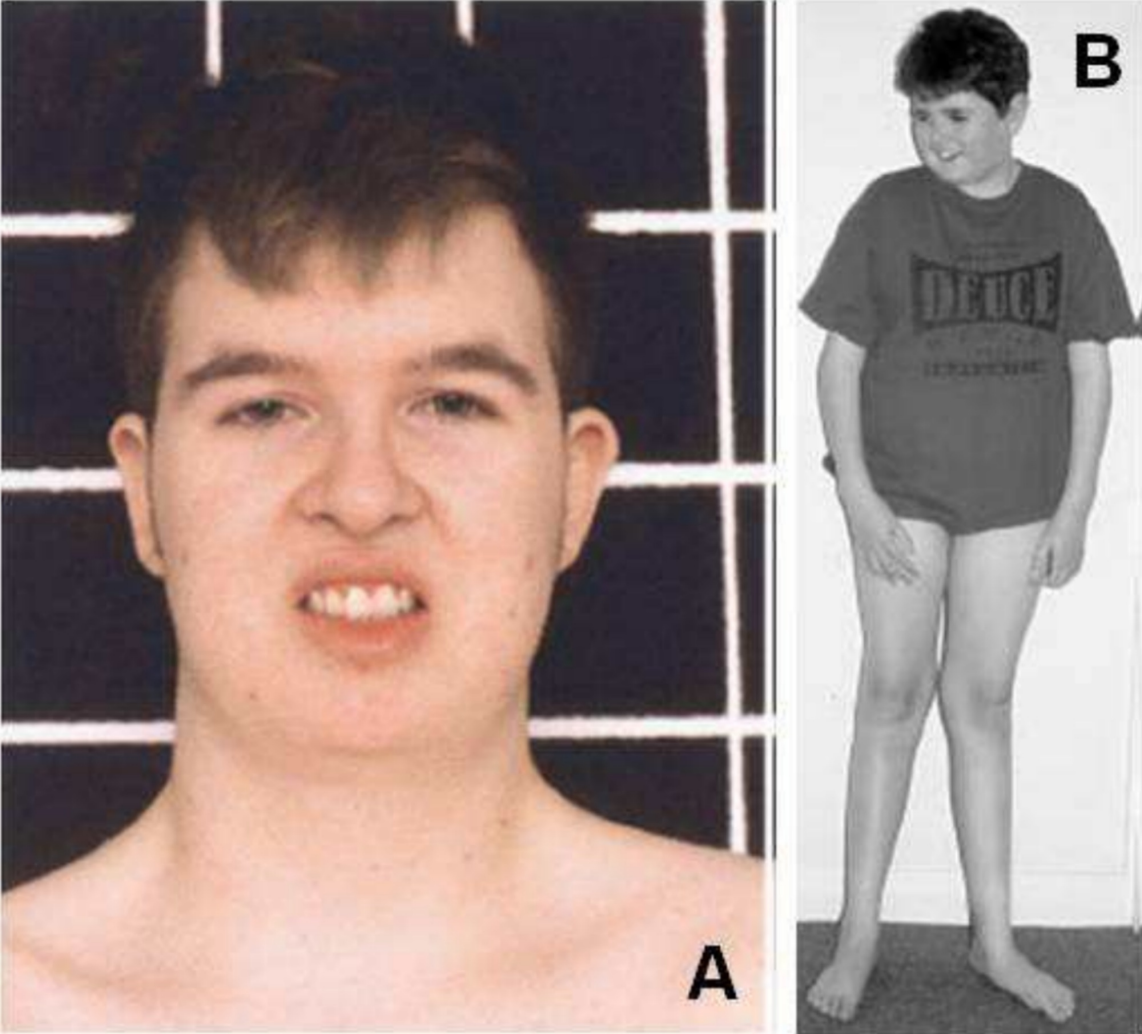
Characteristic somatic features in two males with Cohen syndrome (A) demonstrates downslanting palpebral fissures, thick hair and eyebrows, prominent nose and grimacing expression on smiling. (B) demonstrates truncal obesity and slender extremities

Despite our patient displaying these unique features, his other clinical findings were classic for Cohen syndrome and the diagnosis was made even without a second mutation identified. Our patient met at least two of the three major criteria proposed by Chandler et al., including characteristic facial gestalt and pigmentary retinopathy; neutropenia is being assessed with a pending complete blood count [8]. Other supportive criteria including early-onset, progressive myopia, truncal obesity with slender extremities, and joint hyperextensibility were also met. Figure 4 lists major and minor criteria for Cohen syndrome, and Figure 5 illustrates most of the characteristic somatic features in two males with Cohen syndrome [11]. These figures are useful for both the ophthalmologist and primary care physician in arriving at this diagnosis.

An important aspect of preventing central vision loss in our patient is the treatment of the CME with topical dorzolamide, a carbonic anhydrase inhibitor. Carbonic anhydrase inhibitors have been useful in treating CME in a similar condition, retinitis pigmentosa, and may draw out fluid through altering the polarity of ionic transport systems within the retinal pigment epithelium [12]. Topical nonsteroidal anti-inflammatory agents and topical or injected steroids are other commonly used medications for the treatment of CME [12].

## Conclusions

This case highlights how a high index of suspicion is required to make an early diagnosis and that treatment is critical in preventing total blindness in these patients. Ophthalmologists must consider Cohen syndrome when developmental delay, high-grade myopia, and retinal dystrophy are present in a child. Visual fields, fundus examination and OCT are important diagnostic modalities to identify and monitor for progression of the retinal component. Though treatment is mainly supportive for the disease, the rarely-appearing CME is certainly treatable with medications. This case raises awareness of the syndrome and the need to recognize various clinical features, perform confirmatory genetic testing, and direct appropriate treatment to prevent complications and help improve quality of life.
